# Citrus wastewater as a source of value‐added products: Quali‐quantitative analysis and in vitro screening on breast cancer cell lines

**DOI:** 10.1002/ardp.202400530

**Published:** 2024-10-04

**Authors:** Maria Valeria Raimondi, Salvatrice Rigogliuso, Filippo Saiano, Paola Poma, Manuela Labbozzetta, Marilia Barreca, Marcella Barbera, Roberta Bivacqua, Giovanna Li Petri, Silvestre Buscemi, Ignazio Sardo, Virginia Spanò, Antonio Palumbo Piccionello, Alessandra Montalbano, Paola Barraja, Monica Notarbartolo

**Affiliations:** ^1^ Department of Biological, Chemical and Pharmaceutical Sciences and Technologies (STEBICEF) University of Palermo Palermo Italy; ^2^ Department of Agricultural, Food and Forestry Sciences (SAAF) University of Palermo Palermo Italy; ^3^ Department of Earth and Marine Sciences (DiSTeM) University of Palermo Palermo Italy; ^4^ Istituto per lo Studio dei Materiali Nanostrutturati, CNR Palermo Italy

**Keywords:** antioxidant properties, antiproliferative activity, citrus wastewater, HPLC/MS Q‐TOF analysis, nobiletin

## Abstract

Citrus wastewater from industries is a source of bioactive compounds whose recovery could be a useful approach to convert processing waste into potential resources to be exploited in food, pharmaceutical, and chemical companies. Citrus wastewater, obtained from the industrial processing of *Citrus sinensis*, was freeze‐dried and qualitative/quantitative evaluated using HPLC/MS Q‐TOF analysis. Antiproliferative activity was investigated on MDA‐MB‐231 (triple‐negative breast cancer cell line), MCF‐7 (breast cancer cell line), and its multidrug‐resistant variant MCF‐7R. Fraction **8** emerged for its cytotoxicity toward MCF‐7R cells. Its main component, the polymethoxylated flavone nobiletin (80%), is likely involved in increasing the number of G1‐phase MCF‐7R cells without inducing cell death. Notably, fraction **8** sensitizes MCF7‐R cells to the antiproliferative effects of doxorubicin, thus contributing to overcoming MCF7‐R multidrug resistance. Our studies highlighted the possibility of applying a sustainable strategy for citrus wastewater recycling to recover functional compounds as useful adjuvants for the prevention and treatment of malignancies.

## Graphical abstract

Citrus wastewater obtained from the industrial processing of *Citrus sinensis* proved to be a valuable source of bioactive compounds. Polymethoxylated flavone nobiletin was revealed to be the main component of fraction **8**, which emerged for its cytotoxicity toward MCF‐7R cells and for its ability to sensitize MCF7‐R cells to the antiproliferative effects of doxorubicin.

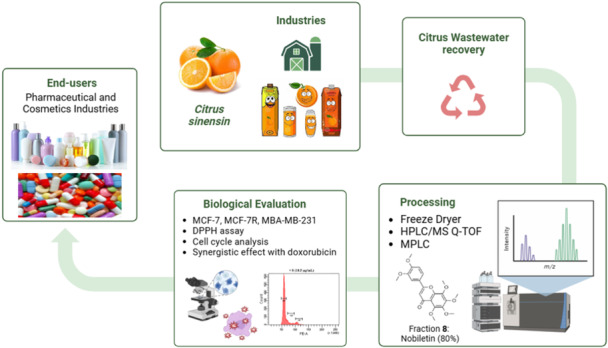

## 1 INTRODUCTION

Citrus fruits represent a valuable source of health‐promoting agents such as phenolic compounds (flavonoids, coumarins, and phenolic acids), essential oils, dietary fibers (pectin), tocotrienols (vitamin E), and many minerals (selenium, copper, zinc, and iron). It is widely known that these bioactive compounds can play a beneficial role against gastrointestinal, coronary, and inflammatory disorders, as well as viral infections and tumor diseases.^[^ ^1^, ^2^ ^]^ Therefore, the identification of new sources of these phytochemicals is highly desirable.

Citrus, which are not used in the market as fresh fruits, are generally industrially processed to obtain juices and essential oils, thus generating both wastewater and by‐products. The latter have recently attracted increasing interest as alternative sources of bioactive components,^[^ ^3^, ^4^ ^]^ with the added ecological value of reducing environmental pollution. In particular, the recovery of functional compounds from wastewater could be exploited in food, pharmaceutical, and chemical companies, ensuring higher conformity to the ecological standards currently requested in the industries. Moreover, the resulting economic advantage would increase the competitiveness of the citrus fruit sector while reducing the incidence of disposal costs.

Citrus wastewater, in particular, is a residue consisting of colloidal dispersion (hesperidin, pectin, etc.), pulp, and peel residues, along with soluble organic compounds (carbohydrates and organic acids) and essential oils. It is characterized by high chemical variability, high acidity, nutrient scarcity, and a moderate concentration of essential oils. Therefore, considering the large wastewater volumes generally produced and their peculiar characteristics, considerable technical‐economic difficulties can be encountered in their sustainable disposal.

Considering that only a few papers have been reported in the literature on the use of citrus wastewater as a source of bioactive compounds,^[^ ^5^, ^6^, ^7^, ^8^ ^]^ herein we investigate the composition of the wastewater obtained from the industrial processing of *Citrus sinensis* from Eurofood. Citrus wastewater was freeze‐dried and subjected to medium‐pressure liquid chromatography (MPLC), and the corresponding fractions thus isolated were analyzed not only in terms of qualitative composition but also for their biological properties. In particular, the antioxidant properties and antiproliferative activities on MDA‐MB‐231 (triple‐negative breast cancer ‐TBN‐ cell line), MCF‐7 (breast cancer cell line), and its multidrug‐resistant (MDR) variant MCF‐7R have been evaluated. Some fractions showed moderate cytotoxic effects against MCF‐7R‐resistant cell line, and therefore, their compositions have been further investigated by HPLC Q‐TOF analysis. Our studies highlighted not only the potential chemoprevention property of selected fractions in in vitro models of breast cancer but also the possibility of applying a sustainable strategy for citrus wastewater recycling to recover functional compounds as useful adjuvants for the prevention and treatment of malignancies.

## 2 RESULTS AND DISCUSSION

### 2.1 Chemical characterization of the citrus wastewater

To investigate the chemical composition of the wastewater, qualitative and quantitative data were collected through HPLC/MS Q‐TOF analysis (Table 1; see also the Supporting Information S1). The mass spectra data revealed the presence of several valuable chemical compounds that were lost in the waste processes, which deserve to be recovered. A total of 35 metabolites were identified, belonging to different classes such as carbohydrates (3 compounds), organic acids (3 compounds), cinnamic acid derivatives (7 compounds), salicylate (1 compound), terpenes (3 compounds), methoxylated flavonoids (9 compounds), non‐methoxylated flavonoids (3 compounds), and limonoids (6 compounds). In particular, citric acid was detected among the components measured in percentages above 1%. Citric acid is widely used in the pharmaceutical, food, and cosmetics industries as well as sanitizers, food preservatives, and acidifiers. It is known for its anti‐scale action along with its ability to reduce water hardness, promote iron absorption, and exert a mild bactericidal and antiarthritic action.^[^ ^9^ ^]^ Other major components are methoxylated flavonoids such as nobiletin and tangeretin, which are endowed with antitumoral activity.^[^ ^10^, ^11^ ^]^

**Table 1:** Metabolite distribution for wastewater by means of HPLC/MS Q‐TOF analysis.

Entry	Compound	Molecular formula	Chemical class	ESI^−^ [M − H]^−^ (*m/z*) (*Calcd*.)	ESI^−^ [M − H]^−^ (*m/z*) (*Found*)	Rt (min)	Area %^a^
1	Glucose	C_6_H_12_O_6_	Carbohydrate	179.0561	179.0569	0.72	0.07
2	Disaccharide	C_12_H_22_O_11_	Carbohydrate	341.1089	341.1081	0.74	0.01
3	Isocitric acid	C_6_H_8_O_7_	Organic acid	191.0197	191.0200	0.87	1.20
4	Citric acid	C_6_H_8_O_7_	Organic acid	191.0197	191.0202	1.05	2.44
5	Homocitric acid	C_7_H_10_O_7_	Organic acid	205.0354	205.0353	1.22	0.23
6	Geranyl diphosphate	C_10_H_20_O_7_P_2_	Terpene	313.0612	313.0604	1.87	0.13
7	Caffeic acid glucuronide	C_16_H_20_O_9_	Cinnamic acid derivative	355.0671	355.0676	2.32	0.04
8	Caffeic acid glucuronide isomer	C_16_H_20_O_9_	Cinnamic acid derivative	355.0671	355.0675	2.83	0.07
9	Caffeoylmalic acid	C_13_H_12_O_8_	Cinnamic acid derivative	295.0488	295.0468	3.35	0.05
10	Salicyl glucuronate	C_13_H_14_O_9_	Salicylate	313.0565	313.0568	3.36	0.09
11	Feruloylgalactaric acid	C_16_H_18_O_11_	Cinnamic acid derivative	385.0776	385.0771	3.76	0.08
12	Coumaric acid glucoside	C_15_H_18_O_8_	Cinnamic acid derivative	325.0929	325.0928	4.46	0.03
13	Feruloylgalactaric acid isomer	C_16_H_18_O_11_	Cinnamic acid derivative	385.0776	385.0772	5.64	0.08
14	Citroside B	C_19_H_30_O_8_	Terpene	431.1923 [M + FA − H]^−^	431.1910 [M + FA − H]^−^	6.06	0.06
15	Anhydroglucose	C_6_H_10_O_5_	Carbohydrate	161.0456	161.0451	8.38	0.03
16	Neohesperidin	C_27_H_30_O_16_	Methoxylated flavonoid	609.1461	609.1437	9.76	0.01
17	Vicenin 2	C_27_H_30_O_15_	Non‐methoxylated flavonoid	593.1512	593.1530	10.84	0.06
18	Naringin glucoside	C_33_H_42_O_19_	Non‐methoxylated flavonoid	741.2248	741.2247	11.21	0.01
19	Diosmetin diglucoside	C_28_H_32_O_16_	Methoxylated flavonoid	623.1618	623.1621	11.69	0.02
20	Limonin‐17‐β‐d‐glucoside	C_32_H_42_O_14_	Limonoid	649.2502	649.2506	12.44	0.28
21	Abscisic acid glucopyranosyl ester	C_21_H_30_O_9_	Terpene	471.1872 [M + FA − H]^−^	471.1874 [M + FA − H]^−^	12.77	0.03
22	Dicaffeoyl quinic acid	C_25_H_24_O_12_	Cinnamic acid derivative	561.1250 [M + FA − H]^−^	561.1235 [M + FA − H]^−^	13.27	0.02
23	Deacetylnomilinic acid glucoside	C_32_H_46_O_15_	Limonoid	669.2764	669.2791	13.90	0.02
24	Naringin	C_27_H_32_O_14_	Non‐methoxylated flavonoid	579.1719	579.1735	14.50	0.10
25	Deacetylnomilin glucopyranoside	C_32_H_44_O_14_	Limonoid	651.2658	651.2686	15.18	0.03
26	Hesperidin	C_28_H_34_O_15_	Methoxylated flavonoid	609.1825	609.1836	15.56	0.12
27	Nomilin glucopyranoside	C_34_H_46_O_15_	Limonoid	693.2764	693.2784	16.64	0.04
28	Nomilinic acid glucoside	C_34_H_48_O_16_	Limonoid	711.2870	711.2885	17.01	0.16
29	Demethylnobiletin	C_20_H_20_O_8_	Methoxylated flavonoid	389.1231 [M + H]^+^	389.1240 [M + H]^+^	25.21	0.76
30	Limonin	C_26_H_30_O_8_	Limonoid	515.1923 [M + FA − H]^−^	515.1936 [M + FA −H]^−^	26.06	0.04
31	Tangeretin	C_20_H_20_O_7_	Methoxylated flavonoid	373.1282 [M + H]^+^	373.1289 [M + H]^+^	26.07	10.27
32	Methoxytangeretin	C_21_H_22_O_8_	Methoxylated flavonoid	403.1387 [M + H]^+^	403.1396 [M + H]^+^	26.47	18.45
33	Nobiletin	C_21_H_22_O_8_	Methoxylated flavonoid	403.1387 [M + H]^+^	403.1398 [M + H]^+^	26.80	39.36
34	Tetramethoxyflavone	C_19_H_18_O_6_	Methoxylated flavonoid	343.1176 [M + H]^+^	343.1195 [M + H]^+^	26.97	14.68
35	Methoxynobiletin	C_22_H_24_O_9_	Methoxylated flavonoid	433.1493 [M + H]^+^	433.1492 [M + H]^+^	27.24	10.95

^a^ Area % obtained from total ion counts (TIC) traces.

The wastewater contains also other substances in lower concentrations that, due to their peculiarities, might be useful to recover. Limonoids are oxygenated terpenoids with interesting biological activities such as anticancer, antimicrobial, antioxidant, antidiabetic, and insecticidal.^[^ ^12^ ^]^

Concerning flavonoids, neohesperidin is used as an intensive low‐calorie sweetener because it has a sweetening power of up to 1800 times more than sucrose.^[^ ^13^ ^]^ Another valuable flavonoid detected in wastewater was hesperidin, used in pharmaceuticals as adjuvant therapy in the treatment of varicose veins, phlebitic complications of hemorrhoids, and those related to capillary fragility.^[^ ^14^, ^15^ ^]^ Moreover, a small amount of naringin (about 0.1%), a flavonoid endowed with several beneficial properties such as antioxidant, anti‐inflammatory, and antiapoptotic, was also recovered. Naringin has liver protective action and is used to mitigate the toxic effects of many drugs by significantly increasing levels of L‐FABP, a protein with protective and antioxidant activity. Indeed, it counteracts the hepatotoxic effects of paracetamol, doxorubicin, and cisplatin.^[^ ^16^ ^]^

Finally, concentrations of non‐methoxylated flavonoids, methoxylated flavonoids, and limonoids in wastewater have also been determined (Table 2).

**Table 2:** Concentrations of non‐methoxylated flavonoids, methoxylated flavonoids, and limonoids in wastewater.

Compound	Concentration (mg/L)
Non‐methoxylated flavonoid	
Vicenin 2	<0.10
Naringin glucoside	<0.10
Naringin	0.27
Limonoid	
Limonin‐17‐β‐d‐glucoside	2.26
Limonin	0.19
Deacetylnomilinic acid glucoside	<0.10
Deacetylnomilin glucopiranoside	0.12
Nomilin glucopiranoside	0.19
Nomilinic acid glucoside	1.24
Methoxylated flavonoid	
Hesperidin	0.43
Neohesperidin	<0.10
Demethylnobiletin	4.19
Tangeretin	43.26
Methoxytangeretin	11.47
Nobiletin	46.95
Tetramethoxyflavone	8.42
Methoxynobiletin	24.35
Diosmetin diglucoside	<0.10

To fractionate the lyophilized components according to their chemical–physical properties, an MPLC has been used. The collected fractions (FRs) were grouped on the basis of the chromatogram output, resulting in eight new fractions subsequently subjected to HPLC/MS Q‐TOF investigation (Table 3; see also the Supporting Information S1) and biological evaluation.

**Table 3:** Main components of separated fractions **1–8**.

FR	Compounds
**1**	Glucose, Disaccharide, Isocitric acid, Citric acid, Homocitric acid
**2**	Geranyl diphosphate, Caffeic acid glucuronide, Caffeic acid glucuronide isomer, Caffeoylmalic acid, Salicyl glucuronate, Feruloylgalactaric acid, Coumaric acid glucoside, Feruloylgalactaric acid isomer, Citroside B, Limonin‐17‐β‐d‐glucoside
**3**	Geranyl diphosphate, Caffeic acid glucuronide, Caffeic acid glucuronide isomer, Salicyl glucuronate, Feruloylgalactaric acid, Feruloylgalactaric acid isomer.
**4**	Citroside B, Vicenin 2, Deacetylnomilinic acid glucoside, Nomilinic acid glucoside
**5**	Naringin, Hesperidin
**6**	Demethylnobiletin, Tangeretin, Methoxytangeretin, Nobiletin
**7**	Tangeretin, Methoxytangeretin, Nobiletin
**8**	Tangeretin, Methoxytangeretin, Nobiletin, Tetramethoxyflavone, Methoxynobiletin

### 2.2 Biological evaluation of the citrus wastewater

#### 2.2.1 Evaluation of the antioxidant properties of the fractions

The antiradical activity of the sample has been evaluated using the diphenyl‐1‐picrylhydrazyl (DPPH) stable radical method. No significant antioxidant activity is detected. Only fractions **2** and **3** showed a moderate antioxidant activity (anti‐free radical capacity ACR 0.024) (Table 4), although not comparable to that of Trolox (pure reference compound). It is interesting to note that, despite the dilution of the wastewater due to the industrial process, a protective action against free radicals, albeit small, can be detected. This result is consistent with the chemical composition of fractions **2** and **3**, in which the main components are well‐known compounds endowed with antioxidant activity.

**Table 4:** Antioxidant activity performed with the DPPH method.

FR	ED_50_ (µg/mL)	(ARC) 1/ED_50_
Trolox	0.65	1.54
**1**	>200	<0.005
**2**	42	0.024
**3**	42	0.024
**4**	84	0.012
**5**	>100	<0.010
**6**	100	0.010
**7**	>100	<0.010
**8**	100	0.010

*Note*: The results are expressed as anti‐free radical capacity (ARC), 1/ED_50_.

#### 2.2.2 Antiproliferative activity of the fractions

The cytotoxic activity of fractions **1–8** was evaluated using MTS assays on all the breast cancer cell lines: MCF‐7, its multidrug variant, MCF‐7R, the TNBC cell line MDA‐MB‐231, and also in a non‐tumorigenic cell line, 1‐7HB2. Cell lines were treated for 72 h with the different fractions using a wide range of concentrations (5–250 µg/mL). As shown in Table 5, the IC_50_ values obtained demonstrate that the different fractions showed cytotoxic activity against all three lines. Most of the fractions (**2**, **4**, **5**, **6**, **7**, and **8**) showed IC_50_ values at micromolar level, against almost all three tumor lines, proving to be potentially exploitable in tumors with different histotypes. On non‐tumorigenic cell line 1‐7HB2, IC_50_ values are generally higher.

**Table 5:** Antiproliferative activity of fractions **1–8** evaluated by MTS assays after 72 h of treatment.

	MCF‐7 cell line	MCF‐7R cell line	MDA‐MB‐231 cell line	1‐7HB2 cell line
FR	IC_50_ (µg/mL) ± SE	IC_50_ (µg/mL) ± SE	IC_50_ (µg/mL) ± SE	IC_50_ (µg/mL) ± SE
**1**	135.0 ± 39.6	168.0 ± 6.0	192.0 ± 4.9	212.5 ± 5.3
**2**	65.5 ± 17.3	75.0 ± 7.0	85.5 ± 0.3	97.5 ± 1.8
**3**	132.0 ± 30.0	143.5 ± 15.0	184.0 ± 0.7	186.5 ± 2.5
**4**	33.5 ± 9.5	43.5 ± 2.5	44.5 ± 2.5	45.5 ± 3.2
**5**	69.0 ± 13.4	81.0 ± 1.4	85.5 ± 0.3	98.5 ± 1.1
**6**	38.0 ± 2.1	43.0 ± 2.1	62.0 ± 4.9	74.0 ± 1.4
**7**	66.0 ± 8.5	38.0 ± 0.7	71.5 ± 3.9	73.5 ± 2.5
**8**	36.0 ± 2.1	18.5 ± 2.5	64.0 ± 4.9	46.5 ± 2.5
Doxorubicin	0.8 ± 0.1	37.0 ± 2.0	0.05 ± 0.004	n.t.

*Note*: Data are expressed as the IC_50_ (concentration inhibiting 50% of cell growth) and are the mean ± standard error (SE) of at least three separate experiments. n.t.: not tested.

Particularly, fractions **7** and **8** proved to be more cytotoxic toward MCF‐7R cells, showing IC_50_ values even lower than the MCF‐7 parental line. This interesting result led us to a deeper investigation of their composition. Therefore, a quantitative analysis of both fractions has been done (Table 6), revealing that the main component of both fractions is the flavonoid derivative nobiletin.

**Table 6:** Main components of fractions **7** and **8**.

FR	Compounds (%)
**7**	Tangeretin (7%), Methoxytangeretin (6%), Nobiletin (87%)
**8**	Tangeretin (3%), Methoxytangeretin (9%), Nobiletin (80%), Tetramethoxyflavone (5%), Methoxynobiletin (3%)

It is well known that this polymethoxylated flavone is endowed with a wide range of pharmacological properties, ranging from anti‐inflammatory and cardioprotective to osteoprotective and antidiabetic.^[^ ^17^, ^18^, ^19^ ^]^ Moreover, nobiletin showed anticancer activity toward different types of human cancer cell lines, such as human colorectal cells (HT‐29), human breast cancer cells (MCF‐7), four gastric adenocarcinoma cell lines (TMK‐1, MKN‐45, MKN‐74, and KATO‐III), and hepatocellular carcinoma cells (SMMC‐7721).^[^ ^20^, ^21^, ^22^, ^23^ ^]^ Finally, it has been reported that nobiletin, in rodents, is able to reduce the development of chemically induced colon carcinogenesis,^[^ ^24^, ^25^, ^26^ ^]^ as well as prostate adenocarcinoma in transgenic rats.^[^ ^27^ ^]^ Many biological activities of nobiletin, as well as inflammatory process inhibition and cell‐cycle arrest or apoptosis induction both in vivo and in vitro, have been suggested in several cancer cells.^[^ ^21^, ^22^, ^23^ ^]^

Both fractions **7** and **8** contain nobiletin as the main component. However, the latter is endowed with a higher antiproliferative activity against all three cell lines probably because the phytocomplex is richer in bioactive compounds.

#### 2.2.3 Effects on cell cycle caused by fraction **8**

Nobiletin and tangeretin cause G_1_ cell cycle arrest but do not induce apoptosis in human breast and colon cancer cells.^[^ ^22^ ^]^ Therefore, the inhibition of proliferation induced by the most active fraction **8**, especially on MCF‐7R cell line, could be the result of cell cycle effects or induction of cell death or a combination of the two events. To analyze the effect on the cell cycle, the MCF‐7R cell line was treated with fraction **8** at the corresponding IC_50_ value of 18.5 µg/mL for 48 h. The induction of cell death and distribution of cells within the cell cycle were analyzed by flow cytometry using propidium iodide.

**Figure 1:**
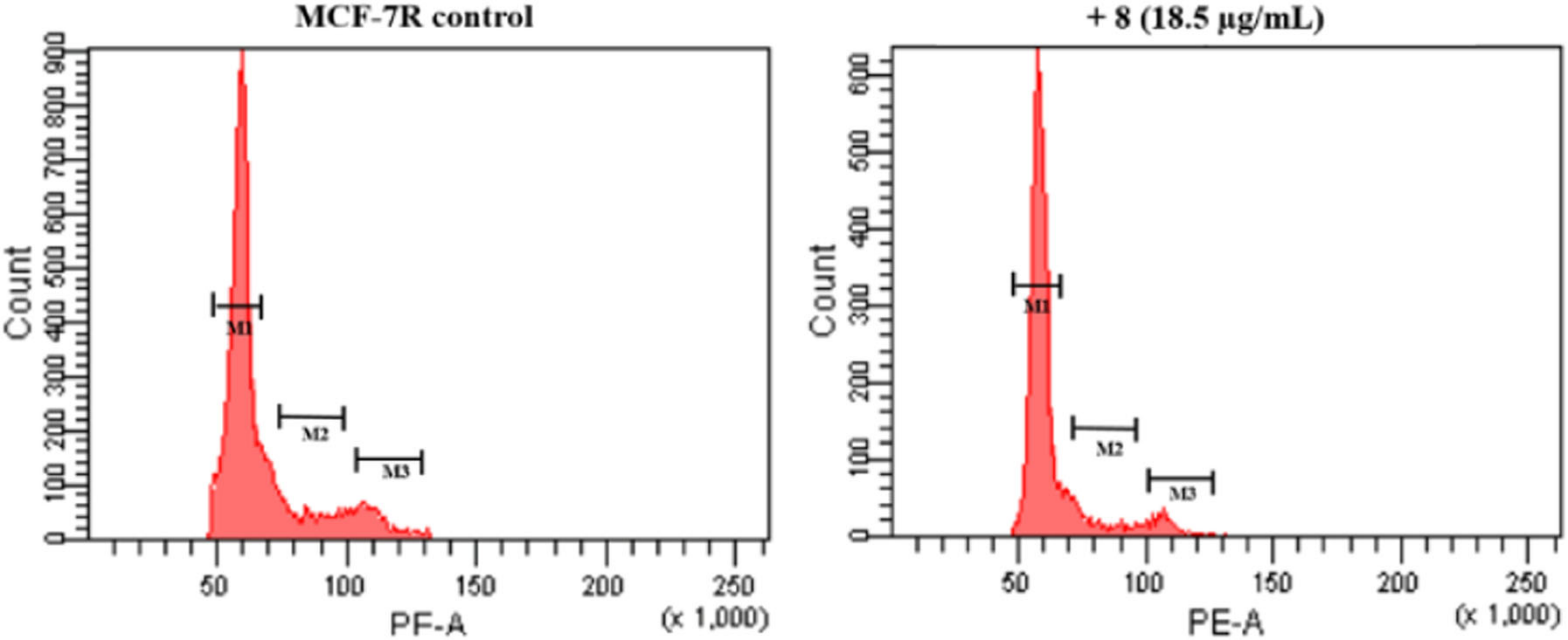
Cell cycle analysis in the MCF‐7R cell line. Cells were treated for 48 h with fraction **8** at the corresponding IC_50_ value of 18.5 µg/mL and their distribution in the phases of the cell cycles was assessed through flow cytometry analysis of their DNA stained with propidium iodide. The panel shows a representative experiment of three independent experiments.

From the cell cycle analysis in MCF‐7R cells emerged that fraction **8** induced a slight increase in the number of G_1_‐phase cells, with a reduction in S‐phase and G_2_/M cells (Figure 1 and Table 7). The cytostatic effect exerted by fraction **8** is in agreement with the data reported in the literature.^[^ ^22^ ^]^

The cells were treated for 48 h with fraction **8** at the corresponding IC_50_ value of 18.5 µg/mL and their distribution in the phases of the cell cycles was assessed through flow cytometry analysis of their DNA stained with propidium iodide. Data are the mean ± S.E. of three separate experiments. *p < 0.01 versus control (one‐way ANOVA followed by Tukey's test).

**Table 7:** Cell cycle changes induced by fraction **8** in MCF‐7R cells.

	G_0_/G_1_	S	G_2_/M
MCF‐7R control	70.4 ± 1.0	15.4 ± 1.0	11.3 ± 0.50
+ FR 8 (18.5 µg/mL)	81.5 ± 0.35*	10.6 ± 0.30*	6.7 ± 0.20*

#### 2.2.4 Antiproliferative effects of the co‐treatments of fraction **8** and doxorubicin

Finally, we wanted to verify if the most active fraction **8** was able to sensitize the MCF‐7R line to the cytotoxic effects of doxorubicin. Sub‐cytotoxic concentrations of fraction **8** and doxorubicin were used. The cytotoxic effects of fraction **8** and doxorubicin, both alone and in combination, were evaluated using MTS assay conducted over 72 h. In Table 8, the cell growth inhibition in percentages obtained by combination versus percentages expected has been reported. The expected percentages are calculated by multiplying the corresponding observed percentages. The data confirmed a considerable enhancement of doxorubicin antiproliferative effects in MCF‐7R cell line (two‐ to threefold). Therefore, fraction **8** showed the interesting ability to sensitize a cell line markedly resistant to doxorubicin to the same chemotherapeutic agent. Since this fraction contains a large amount of nobiletin, we can hypothesize, in line with the results of other authors on ovarian cancer cells, that the potentiation of the antiproliferative effect of doxorubicin is determined by the presence of this compound in the fraction. In fact, nobiletin, as well as verapamil, seems to act by modulating the ATPase activity of P‐glycoprotein (P‐gp) but not its expression in a multidrug‐resistant cell line characterized by the overexpression of P‐gp, as well as our MCF‐7R cells.^[^ ^28^ ^]^

**Table 8:** Results of MTS assay in MCF‐7R cells following 72 h of treatment with fraction **8** and doxorubicin, either alone or in combination.

Treatments	Cell growth inhibition, %	Expected, %
FR **8**: 5 µg/mL	5.0 ± 3.5	
FR **8**: 10 µg/mL	6.0 ± 4.2	
Doxo: 2 µg/mL	10.5 ± 7.4	
Doxo: 5 µg/mL	28.0 ± 0.0*	
FR **8**: 5 µg/mL + Doxo: 2 µg/mL	49.0 ± 0.7**	15.0 ± 3.0*, ^a^
FR **8**: 5 µg/mL + Doxo: 5 µg/mL	59.0 ± 0.7**	31.6 ± 1.0**, ^b^
FR **8**: 10 µg/mL + Doxo: 2 µg/mL	59.5 ± 0.3**	16.0 ± 3.9**, ^a^
FR **8**: 10 µg/mL + Doxo 5 µg/mL	60.0 ± 0.7**	32.0 ± 2.0**, ^a^

*Note*: Data are expressed as the mean ± standard error of three independent experiments.

** *p* < 0.01, versus the control

* *p* < 0.05, versus the control.

^a^ *p* < 0.01, expected versus observed (one‐way ANOVA followed by Tukey's test).

^b^ *p* < 0.05, expected versus observed (one‐way ANOVA followed by Tukey's test).

## 3 CONCLUSION

The aim of our study was the characterization of citrus wastewater to explore the possibility of recovering functional compounds as a useful approach to convert citrus by‐products and processing waste into potential resources to be exploited. Quantitative analysis showed that among the identified metabolites, the most abundant compounds were methoxylated flavonoids nobiletin and tangeretin (Table 2), natural products endowed with several beneficial properties, including antiproliferative activity. Therefore, cytotoxic activity of the fractions obtained from citrus wastewater was evaluated on MCF‐7, MCF‐7R, and MDA‐MB 231 breast cancer cell lines. Above all, fraction **8** emerged for its cytotoxic activity toward MCF‐7R cells, showing IC_50_ values even lower than the MCF‐7 parental line. This interesting activity could be related to the main component of the fraction, which proved to be the polymethoxylated flavone nobiletin (80%). Importantly, our study also revealed the ability of this fraction to sensitize MCF7‐R cell line to the doxorubicin cytotoxicity. Even though further investigations need to be performed to explore the molecular mechanisms responsible for overcoming MCF7‐R multidrug resistance, the results of our study point out the potential use of citrus wastewater for the recovery of valuable bioactive molecules while promoting environmental sustainability.

## 4 EXPERIMENTAL

### 4.1 Chemistry

#### 4.1.1 General

The freeze‐drying process was performed with a ModulyoD Freeze Dryer (Thermo Fisher Scientific). High‐pressure liquid chromatography (HPLC) was carried out on an Agilent 1100 series (Agilent Technologies) using a reversed‐phase C18 column (Zorbax Eclipse Plus Acquity C18‐2.0 × 150 mm^2^, 3 µm) with a Phenomenex C18 security guard column (4 mm × 3 mm). Preparative medium‐pressure liquid chromatographic (MPLC) separation was carried out on a CombiFlash RF200 instrument (Teledyne ISCO) using a reversed‐phase cartridge (Biotage Snap Ultra C18, 25–35 µm). Reagent‐grade solvents, purchased from Carlo Erba or Aldrich, were used for chromatographic separations. Mass spectra were obtained on an Agilent 6540 UHD accurate‐mass Q‐TOF spectrometer equipped with a Dual AJS ESI source applying a potential of 2.6 or 3.5 kV on TIP capillary.

The human breast cancer cell lines MCF‐7 and MDA‐MB‐231 were obtained from ATCC (respectively HTB‐22™ and HTB‐26™). The MDR cell line MCF‐7R was established treating the wild‐type cells with gradually increasing concentrations of doxorubicin. Breast cancer cell lines were cultured in Dulbecco's modified Eagle medium (DMEM) (HyClone Europe Ltd), while 1‐7HB2 cells were cultured in DMEM low glucose supplemented with hydrocortisone (5 µg/mL) and insulin (10 µg/mL). MTS dye (Promega Corporation) was used to perform the cytotoxicity assay, and the absorbance was measured at 490 nm using a microplate absorbance reader (iMark Microplate Reader; Bio‐Rad Laboratories, Inc.). Cell death and cell cycle distribution were performed using a FACSCanto instrument and the data were analyzed using BD FACSDiva software v.6.1.2. (Becton Dickinson).

#### 4.1.2 Preparation of samples

Wastewater of *Citrus sinensis* from Eurofood was filtered using a 0.45‐µm filter and the resulting samples were subsequently treated with calcium hydroxide to remove most of the citric acid (about ≈ 200 mg/L). Then, the freeze‐drying process (ModulyoD Freeze Dryer from Thermo Fisher Scientific) was applied both to concentrate the samples and to prevent the degradation of the compounds dissolved in water. From 10 mL of wastewater, 1 g of lyophilizate was obtained. Samples were stored at −20°C until analysis.

#### 4.1.3 HPLC/MS Q‐TOF analysis

The HPLC experiments were carried out on an Agilent 1100 series. Water and acetonitrile were of HPLC/MS grade, and formic acid was of analytical grade. A reversed‐phase C18 column (Zorbax Eclipse Plus Acquity C18‐2.0 × 150 mm^2^, 3 µm) with a Phenomenex C18 security guard column (4 mm × 3 mm) was used. The flow rate was 0.5 mL/min, and the column temperature was set to 30°C. The eluents were formic acid–water (0.1:99.9, v/v) (phase A) and formic acid–acetonitrile (0.1:99.9, v/v) (phase B). The following gradient was employed: 0–5 min, linear gradient 5% B; 5–15 min, linear gradient 5%–15% B, 15–25 min, linear gradient 15%–30% B, returning to initial conditions in 7 min (5% B) and the injection volume was 25 µL. Mass spectra were obtained on an Agilent 6540 UHD accurate‐mass Q‐TOF spectrometer equipped with a Dual AJS ESI source working in negative or positive mode. N_2_ was employed as desolvation gas at 300°C and a flow rate of 8 L/min. A potential of 2.6 or 3.5 kV was used on the capillary for negative or positive ion mode, respectively. The fragmentor was set to 175 V. Eluate was monitored as total ion counts (TIC) or through a UV detector at 250 nm. MS spectra were recorded in the 150–1000 *m/z* range. For quantitative and semiquantitative analyses Vicenin 2 (as non‐methoxylated flavonoid), Limonin‐17‐β‐d‐glucoside (as limonoid), and Diosmetin (as methoxylated flavonoid) were used as standards. Three stock solutions containing 100 mg/L of each compound were prepared in methanol. Then, other solutions were prepared by successive dilutions with water in the range of 0.1–50 mg/L. A linear relationship between peak area and concentration was observed with a correlation coefficient *R* ^2^ = 0.9896, *R* ^2^ = 0.9978, and R^2^ = 0.9998, respectively, for Vicenin 2, Limonin‐17‐β‐d‐glucoside, and Diosmetin. The minimum detection limit was 0.1 mg/L for all the compounds.

#### 4.1.4 Medium‐pressure liquid chromatography

MPLC separation was performed on a CombiFlash RF‐200 (Teledyne‐Isco) equipped with a Biotage Ultra Snap C18 cartridge. Phases A and B were water and acetonitrile of analytical grade (Sigma‐Aldrich), respectively. The following gradient was employed: 0–5 min, linear gradient 10% B; 5–45 min, linear gradient 10%–40% B, 45–60 min, 40% B, returning to initial conditions in 15 min (10% B). The eluate was monitored through UV absorption at 254 and 280 nm. About 2 g of lyophilizate was dissolved in 2 mL of deionized water and the eluate was collected into 150 FRs (50 mL each), divided into two fraction collector racks (A and B, of 75 tubes each). Fractions have been pooled in eight aliquots, as specified below, and subsequently subjected to HPLC/MS Q‐TOF analysis and biological investigation: **1**. FR 5‐6A; **2**. FR 9A; **3**. FR 19‐22A; **4**. FR 23‐24A; **5**. FR 48‐51A; **6**. FR 20‐29B; **7**. FR 32‐35B; and **8**. FR 36‐48B.

### 4.2 Biological assays

#### 4.2.1 Cell lines and culture conditions

The human breast cancer cell lines MCF‐7 and MDA‐MB‐231 were obtained from ATCC (HTB‐22™ and HTB‐26™, respectively). The MDR cell line MCF‐7R was established treating the wild‐type cells with gradually increasing concentrations of doxorubicin. The MDR variant appears to be resistant to doxorubicin and poorly responsive to some molecules with an antiblastic action. From a molecular point of view, MCF‐7R cells, in addition to being estrogen negative (ER−), are characterized by constitutive activation of the NF‐κB pathway, and consequently by the overexpression of some targets of this transcription factor, such as efflux pump P‐glycoprotein, and IAP proteins (Inhibitor of Apoptosis Proteins) which determine their resistance to drug‐induced cell death. The resistance of the MCF‐7R cell line was evaluated after two exposure passages to doxorubicin (250 ng) with the trypan blue dye exclusion test and the IC_50_ value of doxorubicin in MCF‐7R is approximately 75 times higher than the IC_50_ value obtained in the parental MCF‐7 cell line. MDA‐MB‐231 cell line is characterized by the absence of estrogen receptor (ER−), progesterone receptor (PR−), and HER 2. The triple negative carcinoma is associated with epithelial–mesenchymal transition and a high propensity toward early metastases. Breast cancer cell lines were cultured in DMEM (HyClone Europe Ltd), while 1‐7HB2 cells were cultured in DMEM low glucose supplemented with hydrocortisone (5 µg/mL) and insulin (10 µg/mL). All media were supplemented with 10% heat‐inactivated fetal calf serum, 2 mM l‐glutamine, 100 units/mL penicillin, and 100 μg/mL streptomycin (all reagents were from HyClone Europe). All cell lines were cultured in a humidified atmosphere of 5% CO_2_ at 37°C. Cells with a narrow range of passage numbers were used for all experiments. The cultures were routinely tested for Mycoplasma infection.

#### 4.2.2 2,2′‐Diphenyl‐1‐picrylhydrazyl (DPPH) assay

The DPPH• radical scavenging ability of the samples was measured according to Brand‐Williams et al.^[^ ^29^ ^]^ By reacting the DPPH•, organic nitrogenous radical, with a sample capable of transferring a hydrogen atom or an electron to the radical compound, a discoloration of the solution occurs due to the disappearance of the radical, which can be monitored over time by spectrophotometry at the wavelength of maximum absorption. The antiradical efficiency of the samples was evaluated using the DPPH stable radical method. Thus, 100 μL of the sample (at different dilutions within the linearity range of the assay) was added to aliquots (3.9 mL) of a solution prepared with DPPH (4.8 mg) in methanol (200 mL) and the mixture was incubated for 1 h at room temperature in the dark. The absorbance at 517 nm was then measured using a UV‐Vis spectrophotometer. The initial concentration of DPPH was approximately 60 μM. Lower absorbance values of the reaction mixture indicate higher levels of free radical scavenger activity. The results were reported as the percentage reduction in absorbance at 517 nm [(1 − *A*/*A*_0_) × 100] versus the amount of sample divided by the initial DPPH concentration. Each point was acquired in triplicate. The ED_50_ value corresponds to the micrograms of the sample capable of consuming half the amount of free radicals compared to the micromoles of initial DPPH. The results were expressed as antiradical capacity (ARC), which is the inverse of ED_50_. The scavenging capabilities of the samples on DPPH• radicals were evaluated in comparison with Trolox (6‐hydroxy‐2,5,7,8‐tetramethyl‐croman‐2‐carboxylic acid), the water‐soluble synthetic analog of vitamin E.

#### 4.2.3 Cytotoxicity assay

The breast cancer cells were seeded at 1 × 10^4^/well onto 96‐well plates. After 24 h, the medium was replaced with fresh complete medium, and fractions of wastewater were added at various concentrations. After 72 h, 16 μL of MTS dye (Promega Corporation) was added. The plates were incubated for about 2 h in a humidified atmosphere of 5% CO_2_ at 37°C. The bioreduction of the solution MTS was measured by the absorbance of each well at 490 nm using a microplate absorbance reader (iMark Microplate Reader; Bio‐Rad Laboratories, Inc.). Cytotoxicity was expressed as a percentage (mean ± SE) of the absorbance assessed in the control cells. The expected percentages are calculated by multiplying the corresponding observed percentages.

#### 4.2.4 Cell cycle and cell death analysis

To determine cell death and cell cycle distribution, MCF7R cells (1 × 10^5^) were treated for 48 h with fraction **8** used at its IC_50_ values. After treatment, cells were washed twice with cold PBS and then resuspended at 1 × 10^6^/mL in a solution containing propidium iodide (PI) 50 µg/mL in 0.1% sodium citrate plus 0.03% (v/v) Nonidet P‐40. After about 1 h at 4°C (in the dark) of incubation, the samples were analyzed using a FACSCanto instrument and the data were analyzed with BD FACSDiva software v.6.1.2. (Becton Dickinson). Cell distribution was determined by evaluating the percentage of events accumulated in the different phases of the cycle.

## CONFLICTS OF INTEREST STATEMENT

The authors declare no conflicts of interest.

## Supporting information

Supporting information.

## Data Availability

Data are contained within the article or Supporting Information.
