# Supplementary material for: Citrus wastewater as a source of value‐added products: Quali‐quantitative analysis and in vitro screening on breast cancer cell lines
Source: Arch Pharm (Weinheim). 2024 Oct 4;357(12):2400530. doi: 10.1002/ardp.202400530 (PMC11609902; doi:10.1002/ardp.202400530)
Supplement: Supplementary file 1 — Supporting information. [file ARDP-357-2400530-s001.docx]

Citrus wastewater as a source of value-added products: quali-quantitative analysis and in vitro screening on breast cancer cell lines

Maria Valeria Raimondi^1^, Salvatrice Rigogliuso^2^, Filippo Saiano^3^, Paola Poma^2^, Manuela Labbozzetta^2^, Marilia Barreca^1^, Marcella Barbera^4^, Roberta Bivacqua^1^, Giovanna Li Petri^5^, Silvestre Buscemi^6^, Ignazio Sardo^1^, Virginia Spanò^1^, Antonio Palumbo Piccionello^6,*^, Alessandra Montalbano^1,*^, Paola Barraja^1^ and Monica Notarbartolo^2^

1 Department of Biological, Chemical and Pharmaceutical Sciences and Technologies (STEBICEF), University of Palermo, Via Archirafi 32, 90123, Palermo, Italy

2 Department of Biological, Chemical and Pharmaceutical Sciences and Technologies (STEBICEF), University of Palermo, Viale delle Scienze, Building 16, 90128, Palermo, Italy

3 Department of Agricultural, Food and Forestry Sciences (SAAF), University of Palermo, Viale delle Scienze, Building 4, 90128, Palermo, Italy

4 Department of Earth and Marine Sciences (DiSTeM), University of Palermo, Via Archirafi 20, 90123, Palermo, Italy

5 Istituto per lo Studio dei Materiali Nanostrutturati, CNR, via U. La Malfa 153, 90146 Palermo, Italy

6 Department of Biological, Chemical and Pharmaceutical Sciences and Technologies (STEBICEF), University of Palermo, Viale delle Scienze, Building 17, 90128, Palermo, Italy

*Correspondence:

Prof. Alessandra Montalbano, Department of Biological, Chemical and Pharmaceutical Sciences and Technologies (STEBICEF), University of Palermo, Via Archirafi 32, 90123, Palermo, Italy

Email: alessandra.montalbano@unipa.it

Prof. Antonio Palumbo Piccionello, Department of Biological, Chemical and Pharmaceutical Sciences and Technologies (STEBICEF), University of Palermo, Viale delle Scienze, Building 17, 90128, Palermo, Italy

Email: Antonio.palumbopiccionello@unipa.it


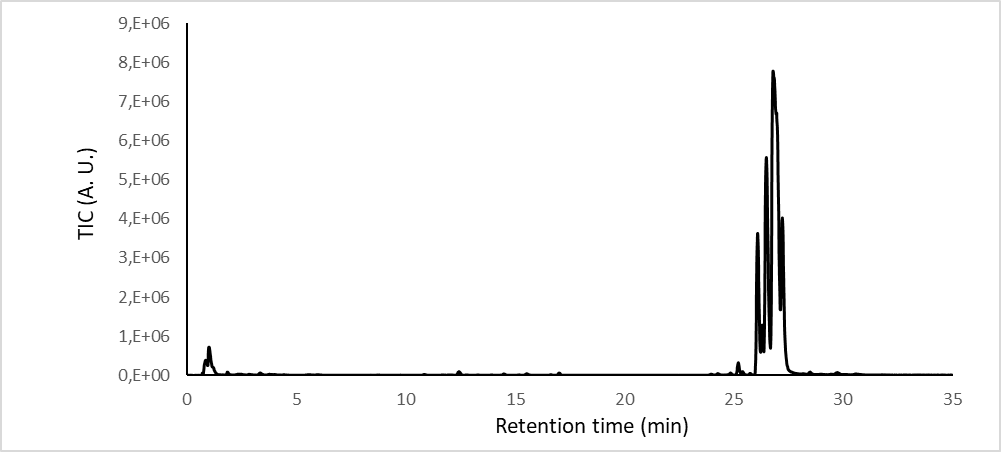


**Figure S1**. HPLC/MS Q-TOF for wastewater monitored as TIC.


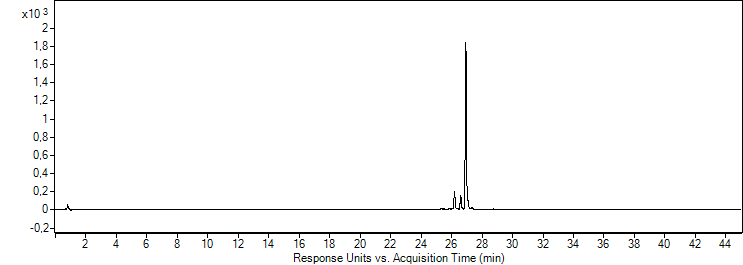


| Peak | RT | Area | Area Sum % |
| --- | --- | --- | --- |
| 1 | 26,19 | 896,18 | 7,19 |
| 2 | 26,6 | 781,32 | 6,27 |
| 3 | 26,93 | 10788,92 | 86,54 |

**Figure S2**. HPLC trace and peaks area of fraction **7**.


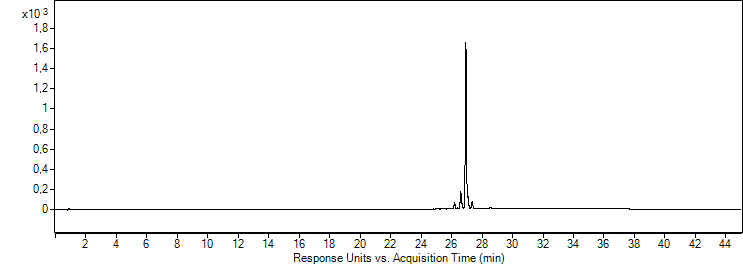


| Peak | RT | Area | Area Sum % |
| --- | --- | --- | --- |
| 1 | 26,2 | 287,09 | 2,53 |
| 2 | 26,6 | 1049,61 | 9,26 |
| 3 | 26,94 | 9035,78 | 79,75 |
| 4 | 27,08 | 594,61 | 5,25 |
| 5 | 27,35 | 362,99 | 3,2 |

**Figure S3**. HPLC trace and peaks area of fraction **8**.
